# Multiscale Network Modeling of Fibrin Fibers and Fibrin Clots with Protofibril Binding Mechanics

**DOI:** 10.3390/polym12061223

**Published:** 2020-05-27

**Authors:** Sumith Yesudasan, Rodney D. Averett

**Affiliations:** 1 Department of Engineering, University of Jamestown, Jamestown, ND 58402, USA; sumith.yd@uga.edu; 2 School of Chemical, Materials, and Biomedical Engineering, College of Engineering, University of Georgia, Athens, GA 30602, USA

**Keywords:** fibrinogen, fibrin, mechanics, multiscale modeling, extracellular matrix, blood clot

## Abstract

The multiscale mechanical behavior of individual fibrin fibers and fibrin clots was modeled by coupling atomistic simulation data and microscopic experimental data. We propose a new protofibril element composed of a nonlinear spring network, and constructed this based on molecular simulations and atomic force microscopy results to simulate the force extension behavior of fibrin fibers. This new network model also accounts for the complex interaction of protofibrils with one another, the effects of the presence of a solvent, Coulombic attraction, and other binding forces. The network model was formulated to simulate the force–extension mechanical behavior of single fibrin fibers from atomic force microscopy experiments, and shows good agreement. The validated fibrin fiber network model was then combined with a modified version of the Arruda–Boyce eight-chain model to estimate the force extension behavior of the fibrin clot at the continuum level, which shows very good correlation. The results show that our network model is able to predict the behavior of fibrin fibers as well as fibrin clots at small strains, large strains, and close to the break strain. We used the network model to explain why the mechanical response of fibrin clots and fibrin fibers deviates from worm-like chain behavior, and instead behaves like a nonlinear spring.

## 1. Introduction

Constitutive modeling of fibrin fibers and fibrin clots is still a challenging topic and is necessary for understanding the origin of the mechanical properties under various loading conditions. Despite advancements in experimental methods to determine the molecular composition and crystal structure of fibrinogen, a major constituent of blood clots, our understanding of fibrin clot mechanics remains poorly understood, and is crucial in developing thrombolytic therapies and diagnosis of thrombosis [1]. In the past, researchers have utilized both the three-chain model and eight-chain model to determine the constitutive response of fibrin clots [2]. Although these models provide benefits for modeling small-strain behavior, the results from these studies show inadequacies at fully representing the constitutive behavior of fibrin clots at higher strains [2]. In this paper, we present a nonlinear network model, which was then employed to construct a constitutive model of fibrin fibers, and eventually a fibrin clot at the macroscale.

Fibrin clot polymerization modeling [3,4] and fibrin mechanics [2,5,6] are important research topics for the scientific community. Among these methods, the eight-chain model of fibrin fibers [2] and fibrin clots shows good agreement with past experimental results [7]. This model assumes that the fibrin fibers are composed of a linear elastic component and a worm-like chain (WLC) component, accounting for fibrinogen unfolding at the macroscopic scale. However, results from this model do not match well with experimental results at large strain values. To address this issue, we constructed the fibrin protofibril element using a network model derived from phenomenological modeling of fibrinogen based on atomistic simulation results and the D–E region interaction forces of rupture from atomic force microscopy (AFM) experiments. The proposed nonlinear network model was successful at capturing the force-–extension relationship of fibrin fibers at all strain levels. The validated fiber model was then combined with the modified version of the Arruda–Boyce eight-chain model [8] and applied to a cylindrical shaped fibrin clot under stretching. The force–extension results match well with the experimental results for small and large strain values.

## 2. Multiscale Model of Fibrin Fibers

Fibrin clot formation initiates in the event of thrombosis or hemostasis. Fibrinogen molecules are converted to fibrin monomers through action of the enzyme thrombin. Fibrin monomers polymerize predominantly through knob-hole (A–a) interactions, and enhance connectivity through γ–γ bonding, and weaker bonding through α–α and α–γ interactions. These interactions are stronger in the presence of Factor XIIIa and form dimers, trimers, oligomers, and eventually double stranded protofibrils, which then crosslink with each other to form fibrin fibers, and subsequently a fibrin network. Detailed explanations related to fibrin polymerization and molecular level details can be found in the literature [9,10,11]. More details about the fibrin polymerization process and associated coarse grain modeling procedures can also be found in the literature [3,4,12].

**Figure 1:**
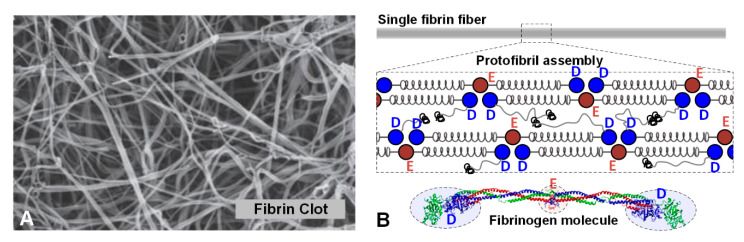
(**A**) Scanning electron microscopic image of a human fibrin clot [13]. (**B**) Multiscale arrangement of a fibrin fiber. Hierarchical arrangement of fibrin(ogen) assembling into protofibrils, and lateral aggregation to form fibers is shown schematically. A molecular model of the fibrinogen molecule is shown below with the D-region and E-region highlighted.

A typical fibrin clot, as observed from scanning electron microscopy (SEM) imaging, is shown in Figure 1A. A schematic showing the physical construction of a single fibrin fiber for modeling purposes is shown in Figure 1B. The fibrinogen molecular model, double stranded protofibril and sample inter-connectivity, and a mature fibrin fiber are also observed in Figure 1B (bottom). The construction of the protofibril strand, from a mechanics perspective, was modeled using a mechanical spring network. The goal of this work was to construct the biopolymer complex into a nonlinear network of mechanical entities, whose properties were estimated from atomic force microscopy (AFM) measurements or from molecular dynamics (MD) simulations.

This paper is organized into the following sections for convenience of the reader. Three main models were developed to simulate the mechanical behavior of a fibrin clot. Model 1, the fibrinogen model, provides a representation of the fibrinogen molecule under stretching based on MD simulation results. Model 2, constructed from Model 1 and with additional protofibril interactions, was used to simulate the mechanical behavior of a single fibrin fiber. Model 3, which represents a fibrin clot, is a multiscale model and consists of Model 2 and Model 1 used in conjunction with the Arruda–Boyce eight-chain model. In summary, Model 1 was employed for modeling the single fibrinogen molecule, Model 2 represents the model of a single fibrin fiber strand, and Model 3 was used to model multiple fibrin fibers (fibrin clot at the macroscale) under stretching.

## 3. Model 1—Fibrinogen Model Development (Atomic Scale)

The force–extension relationship of a single fibrinogen molecule is available from both atomic force microscopy (AFM) experiments [7,14] as well as from molecular dynamics (MD) simulations [7,14,15]. Data for the MD simulation trajectories were obtained from previous analyses. The mechanical response of the individual fibrinogen molecule was obtained from [14], where five total MD simulations were conducted to obtain the steered molecular dynamics (SMD) trajectory data.

Often, the AFM results for the fibrinogen force relationship do not show a consistent trend [7]. Hence, we used the results from the MD simulations of fibrinogen under stretching. The force–extension relationship of the fibrinogen molecule from MD simulations shows a nonlinear response [7]. This nonlinear force-extension relation is a combination of a linear spring and a cubic nonlinear spring (Model 1, Equation (1)).

(1) $$F_{fibrinogen}=k_{1}x+k_{2}H\left[x-d_{2}\right]{\left(x-d_{2}\right)}^{3}$$

**Figure 2:**
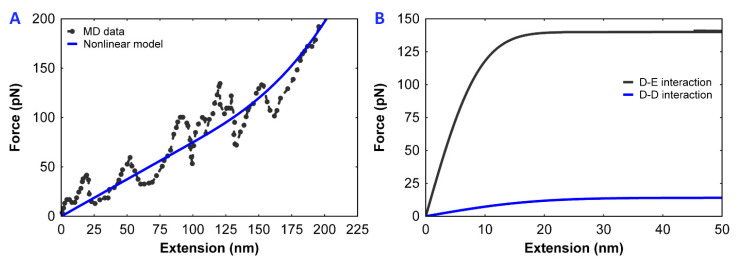
(**A**) Force–extension comparison of a single fibrinogen molecule from molecular dynamics (MD) simulation data [7] and from our nonlinear model. (**B**) Force–extension curve used to simulate the γ–γ interaction and knob–hole (A–a) interactions and their detachment.

Here, $k_{1}$ is the linear spring constant, $k_{2}$ is the nonlinear spring constant, x is the extension between the end-to-end D-region of a single fibrinogen molecule, $d_{2}$ is the distance at which nonlinearity takes effect, and H is the Heaviside function. The resulting profile is shown in Figure 2A, with a correlation coefficient of 0.96. The corresponding parameters are provided in Table 1. The parameter $d_{2}$ was taken as 46 nm, the length of the fibrinogen molecule, in order to relate to the unfolding of the β-structure, which occurs around 150–300 pN [16,17], and $k_{1}$ and $k_{2}$ were adjusted to match the results.

## 4. Modeling of the Protofibril Element—Model 2 (Mesoscale)

**Figure 3:**
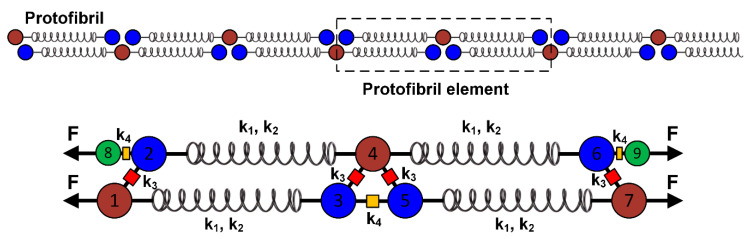
Development of a protofibril element using the nonlinear spring network. A portion of the double stranded protofibril (top) was considered in the development of the nonlinear protofibril element (bottom). Numbering of D- and E-regions from 1 to 9 is for convenience, where k_1_ and k_2_ represent spring constants. Constants k_3_ and k_4_ are components of the force coupling equations described in the manuscript. **F** is the applied force required to extend the protofibril.

The primary and most important component of the protofibril model is the fibrin monomer or the fibrinogen molecule itself. The fibrinogen molecule is a hexamer composed of three pairs of polypeptide chains α, β, and γ (blue, green, and red chains, respectively, in Figure 1B). The crystal structure and molecular construction details of the human fibrinogen molecule are available from the RCSB (PDB entry 3GHG) and have been discussed in the literature [18]. Consider Figure 3, in which a portion of the long protofibril (top) and a conceptual element that has been constructed (bottom) are shown. The C-terminals of the β and γ chains form nodules (D-region) at both ends of the molecule (Figure 3). The N-terminals form to comprise the central E-region.

The fibrinogen molecule can be considered as a combination of two springs connected in series (Model 1, Figure 3, sample molecule is 2-4-6 node sequence). The connection between the D-region and the E-region of different fibrin monomers was modeled using a nonlinear force–extension relationship (k_3_ (red), Figure 3). The γ–γ interaction (3–5 or D–D interaction) was also considered in the model (k_4_ in Figure 3). In Figure 3, nodes 2, 3, 5, and 6 correspond to the D-region; 1, 4, and 7 correspond to the E-region; and nodes 8 and 9 correspond to the dummy nodes for force calculation.

The schematic of the protofibril element is shown in Figure 3, and is assumed to be in quasi-static equilibrium. The free body diagram shows the force balance of the element with F as the force induced per fibrin monomer.

The interaction of the D-region with the E-region (k_3_ components in Figure 3) was considered as a relatively weak hydrophobic attraction known as the knob–hole interaction (A–a), and was modeled using an error function (Equation (2)). From the literature, it is known that the force required to break one of the interactions ($k_{3}$) is in the range of 130–140 pN [17,19]. Equation (2) mathematically represents the distance dependent force between the D- and E-regions of fibrinogen. A smaller value of $d_{3}$ reduces the spread of the function, corresponding to tighter covalent bonding and a larger value simulates plastic mechanical behavior. As no experimental data were available to estimate the flexibility of A–a interactions, we assumed that the maximum elongation that D–E coupling undergoes before rupturing was 10 nm ($d_{3}$).

(2) $$F_{D-E}=k_{3}erf\left(x/d_{3}\right)$$

The γ–γ interaction between the D regions was considered weak as compared to the D–E interaction and was modeled using Equation (3). As no quantified experimental data related to the rupturing force of D-dimers were available, we assumed a similar order of magnitude of B–b knob–hole interactions [20]. Thus, $k_{4}$ is 10 times weaker than $k_{3}$ and $d_{4}$ is twice as large as $d_{3}$.

(3) $$F_{D-D}=k_{4}erf\left(x/d_{4}\right)$$

Equations (2) and (3) (Figure 2B) display an initial linear resistance and later lead to plastic mechanical behavior and elongation.

### 4.1. Solution Procedure

Figure 3 shows the protofibril element with nodes 1 to 7. For every node, force balance conditions were performed under quasi-static loading to derive the system of nonlinear force–extension equations (see Appendix A). This process was extended for the entire length of the protofibril. For reference and comparison, we used similar parameters as in Liu et al. [21]. The length of the fiber was 12 μm, the diameter was 330 nm, fiber protein content was established as 30%, and the fibrin monomer radius was 2.25 nm. This corresponded to 1,614 monomers per cross-sectional area and 261 protofibril elements along the length of the fiber. As a first consideration, the protofibrils were all parallel and not interconnected. This resulted in a fibrin fiber force of $F_{fiber}=1614\times F$, where F is the force per fibrin monomer.

**Figure 4:**
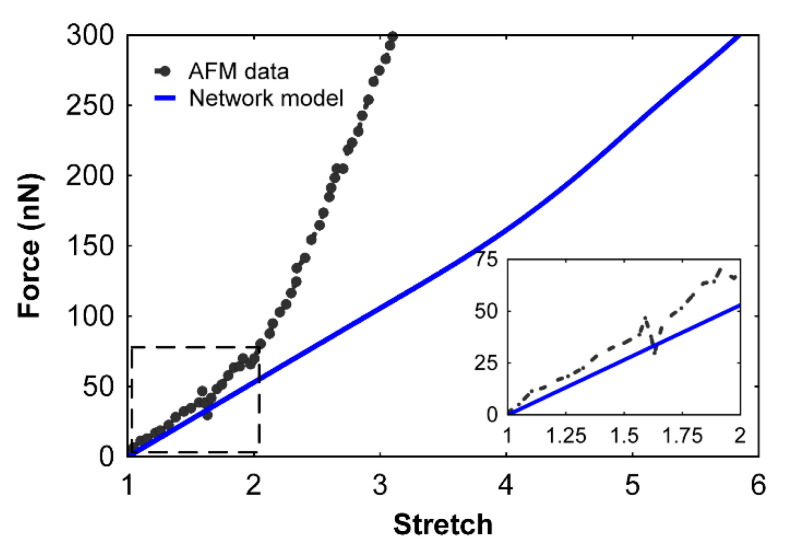
The force–stretch relationship comparison of a 12 μm long, 330 nm diameter fibrin fiber from atomic force microscopy (AFM) experiments [16] (dotted line) and our network model (blue). The inset shows the zoomed in location of the curve with good agreement in the linear region.

The system of nonlinear equations was deduced and can be considered as a nonlinear minimization problem. The Jacobian for this system is sparse and singular, which provided difficulty in solving using standard methods such as the Levenberg–Marquardt algorithm [22]. Other standard implementations in MATLAB [23] and utilization of the Trust Region Dogleg algorithm [24] provided good stability. Force, F, was applied at 500 steps, beginning from 0 pN and reaching a maximum of 200 pN. The resulting force–extension relationship is shown in Figure 4, compared with the results from AFM experimental data [21]. The comparison shows good agreement in the initial stage (inset of Figure 4) and later divergence, showing a less stiff fiber network model. This result is not surprising because, at this stage, we have not considered the effects of inter-protofibril attraction, α-C chain cross linking effects, and effects of the presence of water solvent.

### 4.2. Protofibril Binding Force

It is important to include the effects of inter-protofibril lateral interactions to account for the accurate mechanical properties of fibrin fibers. The primary contributors to this interaction are α-C chain cross linking, binding of Factor XIIIa, presence of water around and inside fibrin fibers, and inter-protofibril hydrophobic and Coulombic attractions. This leads to a complex system and necessitates atomistic details of these individual interactions, which are currently unavailable in the literature. However, this can be achieved by considering a unified force of binding between the protofibrils that participate in the event of force application. An ideal choice for this type of binding force is similar to the nonlinear model that was used for the fibrinogen molecule. This includes a linear component and a distance-dependent cubic nonlinear component. The nonlinear spring constant was coupled to the linear constant by a factor to reduce the number of unknowns. The result is provided in Equation (4):

(4) $$F_{Binding}=k_{5}x_{elem}+k_{6}H\left[x_{elem}-d_{5}\right]{\left(x_{elem}-d_{5}\right)}^{3}$$

**Figure 5:**
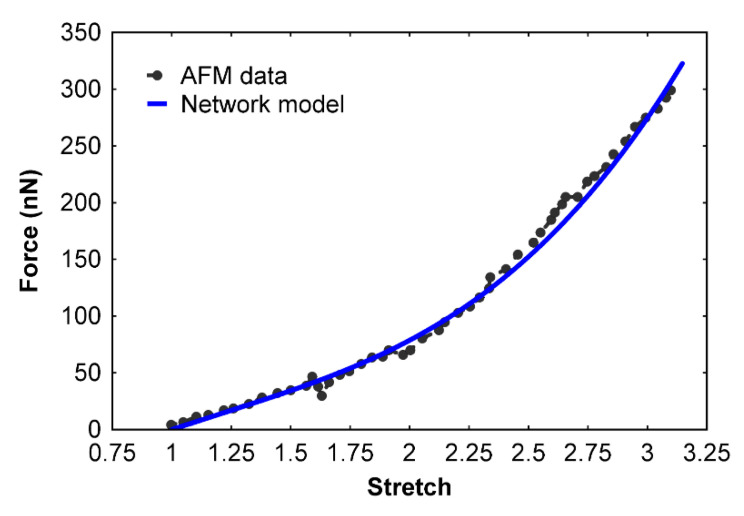
Fibrin fiber force–stretch relationship of our new multiscale network model (Model 2) compared with atomic force microscopy data [21]. The network model (Model 2) accounts for inter-protofibril binding forces.

Here, $k_{5}$ is the linear stiffness of the spring, $k_{6}$ is the nonlinear stiffness, $x_{elem}$ is the elongation of a single protofibril element, and $d_{5}$ is the distance at which the nonlinear spring takes effect. On the basis of our simulations, the parameters were found to be $k_{5}=0.1\;pN/nm$, $k_{6}=\frac{k_{5}}{500}\;pN/nm^{3}$, and $d_{5}=\frac{L_{elem}}{4}\;nm=11.5\;nm$. Using these parameters, Equation (4) was connected across the protofibril element acting in parallel with the remaining network. A value of 0 pN/nm for $k_{5}$ switches off the effect of this binding force in the element. The resulting force–extension curve of the network model was compared with the AFM experimental data (Figure 5). Stretch is defined as the ratio of the deformed fiber length to the original length. The results show good agreement with the AFM data up to the break strain limit.

The parameters used for the various equations used in this work are summarized in Table 1.

### 4.3. Sensitivity of the Force–Extension Curve

**Figure 6:**
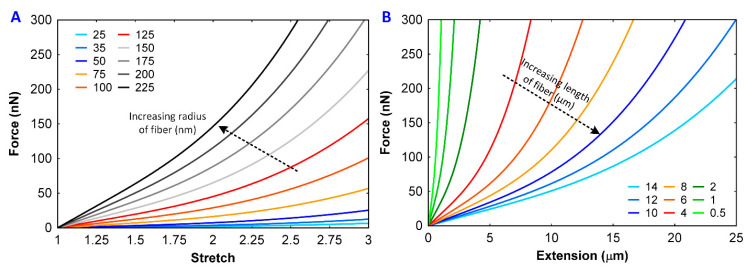
Force–extension curves of fibrin fibers and sensitivity with the (**A**) diameter of the fibrin fibers and (**B**) length of the fibers.

To test the model sensitivity with the radius and length of the fibers, the radius of the fibrin fiber was changed from 25 nm to 225 nm and the length was maintained at 12 μm. Next, the radius was kept constant at 165 nm and the length of the fibrin fiber was varied from 0.5 μm to 14 μm. The resulting force–extension curves are shown in Figure 6A,B. The trend of the curves shows steeper slopes for the increasing diameter of the fibers (Figure 6A) and steeper curves for the decreasing length of the fibers (Figure 6B).

## 5. Modeling of Fibrin Clot—Model 3 (Macroscale)

The next step was to apply the mesoscale network model (Model 2) to a continuum level fibrin clot. Previously, Brown et al. [2] performed stretching on a 2 mm diameter fibrin clot and developed a constitutive model to describe its mechanical behavior. However, the model did not correlate well with the experimental results at higher stretch values. Thus, we extended our fiber network model (Model 2) to this fibrin clot system using the eight-chain model originally developed by Arruda and Boyce [8]. With the assumption of incompressibility, the force–extension relationship of a fibrin cylindrical clot under uniaxial extension takes the form as shown in Equation (7). If the principal stretch, $\lambda_{1}$, is aligned to the axis of extension, then the incompressibility condition leads to the following relation:

(5) $$\lambda_{2}=\lambda_{3}=1/\sqrt{\lambda_{1}}$$

From the eight-chain model, this leads to the stretch of the internal fiber as follows:

(6) $$\lambda_{fiber}=\sqrt{\frac{\lambda_{1}^{2}+2/\lambda_{1}}{3}}$$

The fiber–stretch relationship from (6) along with the force–extension relationship deduced from the network model, as explained in the previous section, leads to the following force-extension relationship for a fibrin clot:

(7) $$F_{clot}=\left(\lambda_{1}-\frac{1}{\lambda_{1}^{2}}\right)\frac{\pi D^{2}vL_{fiber}}{24\lambda_{fiber}}F_{fiber}$$

Here, $F_{fiber}$ is the force–extension relationship of the fibrin fiber deduced from the network model; $\lambda_{1}$ is the principal stretch aligned with the axis of uniaxial extension; D is the diameter of the fibrin clot, taken as 2 mm; v is the fiber density, taken as 0.5 μm^−3^; and L is the length of the fibrin fiber. The parameters in Equation (7) are the same as those in the literature [2], and the value for $F_{fiber}$ is the same as in Table 1, for consistency. The relation between force and extension of the fibrin clot as in Equation (7) is referred to as Model 3 (Macroscale Model).

### Comparison with Fibrin Clot Experiments

**Figure 7:**
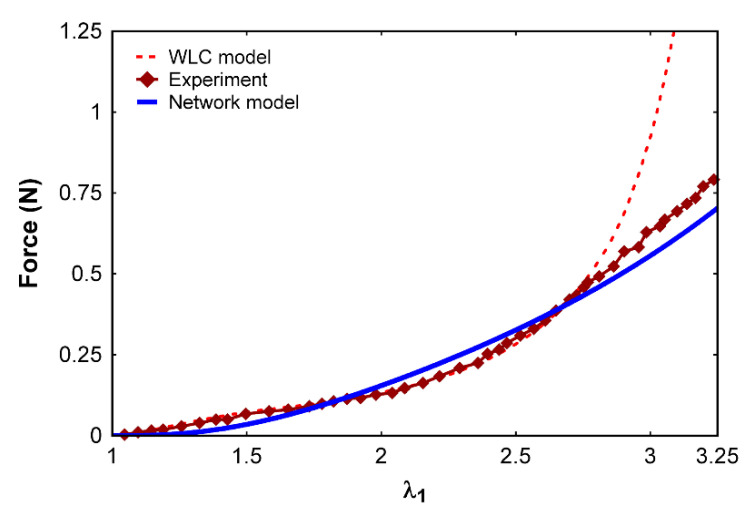
Comparison of the force–stretch relationship for a fibrin clot using our network model with experimental data [2] and with the worm-like chain (WLC) model [2].

The resulting fibrin clot extension data were compared with the experimental results, as well as with the previously available worm-like chain (WLC) model (Figure 7).

The results from Figure 7 show that our network model captures the mechanical behavior of fibrin clots when compared to experiments. While the WLC-based model deviates from experimental results at higher stretch values, our network model provides good agreement. This is mainly because the fibrin fibers and clots behave as a cubic nonlinear material rather than behaving purely like a WLC. It holds true that, as a stiffer entity, DNA can be modeled as a WLC [25]; however, in the case of fibrinogen, the AFM data [7] and molecular simulations [7] show a nonlinear behavior that does not correlate well with the WLC model alone. From the experimental results, fibrin fibers (Figure 5) and fibrin clots (Figure 7) do not behave exclusively as a WLC. Instead, the mechanical behavior is exhibited that is a combination of both linear and nonlinear entities, as suggested by our network model. In the case of WLC models, after the chains stretch closer to the maximum length, a stiffer mechanical response is observed. In the case of fibrin fibers, after stretching more than twice its original length, the force requirement increases for similar stretches, suggesting that the behavior is the result of the unfolding of fibrinogen molecules as well as the sliding of the protofibrils that initiate, ultimately resulting in the nonlinear trend.

## 6. Assumptions and Future Improvements

**Figure 8:**
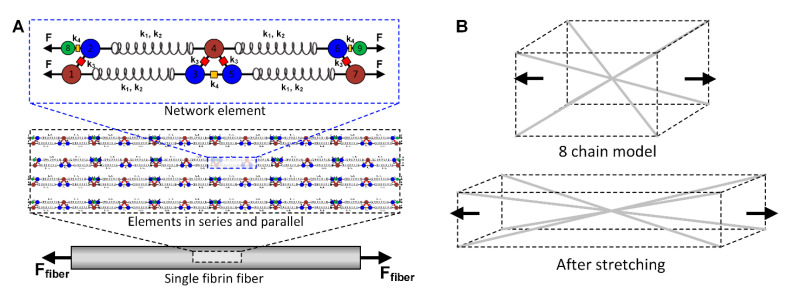
(**A**) Arrangement of network model element (Model 2) in series and parallel to form a fibrin fiber subjected to an externally applied force. (**B**) Schematic of the eight-chain model (Model 3) used for fibrin clot modeling (the diagonal connecting lines represent the fibrin fibers).

Currently, the model assumes that a single fibrin fiber is constructed from a cluster of network elements, forming long protofibrils of length 12 μm, as shown in Figure 8A. Physiologically, this is not the case, but instead, the individual protofibril is composed of up to 15–20 fibrin monomers [26], reaching a length approximately 0.5 μm. Splitting this into protofibril fragments will lead to modeling of the entire fiber and the ensuing complex interconnectivity.

At the molecular scale, it is known that hydrophobic physical interactions are short-ranged; however, this does not imply that the D–E interactions are as short-ranged. Interactions between protein regions or domains are often, if not always, affected by electrostatics, which are much longer ranged. As an improvement to the current study, a sensitivity analysis could be performed on the *d_3_* parameter by varying it to determine the robustness in the selection of the 10 nm value.

At the macroscale, the eight-chain model by Arruda–Boyce and its conceptual model are displayed in Figure 8B. The fibrin fibers progress from the corners of a rectangular volume and eventually connect at the center. When the entire volume is subjected to an external stretch or force, the internal fibers also get strained, as shown in Figure 8B. In the original eight-chain model, the researchers used a strain energy density function derived from the statistical behavior of the chains. In our work, we modified this function and replaced the fiber force–extension relationship with our nonlinear network model. In addition, utilizing stochastic techniques as in [27] can provide an initial random fiber arrangement, prior to force application on the fibrin network.

## 7. Conclusions

In this work, a multiscale network model for fibrin fibers (Model 2) and fibrin clots (Model 3) was developed that is suitable for incorporation into standard continuum level models. The protofibril element (Model 2) was constructed based on molecular simulations and AFM data to simulate the force–extension behavior of fibrin fibers. With the interaction of protofibrils, and with solvent and other binding forces incorporated, it was determined that the force–extension behavior of a single fibrin fiber correlates strongly with experimental results. The validated fibrin fiber network model was then combined with the classical eight-chain model to form a multiscale network model (Model 3), and the force–extension behavior of the continuum level fibrin clot was estimated and shows good correlation. The results show that this network model was successful at predicting the mechanical behavior of fibrin fibers as well as a fibrin clot even when the extension was close to the break strain. We also explained the rationale of why fibrin clots and fibrin fibers deviate from WLC behavior, as in the case of a fibrinogen molecule, using our network constitutive model.

## Figures and Tables

**Table 1:** Parameters and corresponding values used in this study.

Parameter	Unit	Value	Parameter	Unit	Value
$k_{1}$	pN/nm	1.5	$d_{2}$	nm	46
$k_{2}$	$\mathrm{pN}/{\mathrm{nm}}^{3}$	0.0003	$d_{3}$	nm	10
$k_{3}$	pN	130	$d_{4}$	nm	20
$k_{4}$	pN	13	$d_{5}$	nm	11.5
$k_{5}$	pN/nm	0.1			
$k_{6}$	$\mathrm{pN}/{\mathrm{nm}}^{3}$	2 × 10^–4^			

## Appendix A. Mathematical Formulation of Network Element

The force balance of the protofibril element shown in Figure 3 at every node is given here.

(A1) $$N_{1}:k_{3}erf\left(\frac{\left(x_{2}-x_{1}\right)}{d_{3}}\right)+k_{1}\left(x_{3}-x_{1}\right)+k_{2}H\left(x_{3}-x_{1}-d_{2}\right){\left(x_{3}-x_{1}-d_{2}\right)}^{3}-F=0$$

(A2) $$\begin{array}{cc} N_{2}:\;k_{1}\left(x_{4}-x_{2}\right) & +k_{2}H\left(x_{4}-x_{2}-d_{2}\right){\left(x_{4}-x_{2}-d_{2}\right)}^{3}-2k_{3}erf\left(\frac{\left(x_{2}-x_{8}\right)}{d_{3}}\right)\\ & -k_{3}erf\left(\frac{\left(x_{2}-x_{1}\right)}{d_{3}}\right)=0 \end{array}$$

(A3) $$\begin{array}{c} N_{3}:k_{4}erf\left(\frac{\left(x_{5}-x_{3}\right)}{d_{4}}\right)+k_{3}erf\left(\frac{\left(x_{4}-x_{3}\right)}{d_{3}}\right)-k_{1}\left(x_{3}-x_{1}\right)\\ -k_{2}H\left(x_{3}-x_{1}-d_{2}\right){\left(x_{3}-x_{1}-d_{2}\right)}^{3}=0 \end{array}$$

(A4) $$\begin{array}{cc} N_{4}:k_{1}\left(x_{6}-x_{4}\right) & +k_{2}\text{Η}\left(x_{6}-x_{4}-d_{2}\right){\left(x_{6}-x_{4}-d_{2}\right)}^{3}-k_{1}\left(x_{4}-x_{2}\right)\\ & +k_{2}\text{Η}\left(x_{4}-x_{2}-d_{2}\right){\left(x_{4}-x_{2}-d_{2}\right)}^{3}+k_{3}erf\left(\frac{\left(x_{5}-x_{4}\right)}{d_{3}}\right)\\ & -k_{3}erf\left(\frac{\left(x_{4}-x_{3}\right)}{d_{3}}\right)=0 \end{array}$$

(A5) $$\begin{array}{cc} N_{5}:k_{1}\left(x_{7}-x_{5}\right) & +k_{2}H\left(x_{7}-x_{5}-d_{2}\right){\left(x_{7}-x_{5}-d_{2}\right)}^{3}-k_{3}erf\left(\frac{\left(x_{5}-x_{4}\right)}{d_{3}}\right)\\ & -k_{4}erf\left(\frac{\left(x_{5}-x_{3}\right)}{d_{4}}\right)=0 \end{array}$$

(A6) $$\begin{array}{c} N_{6}:k_{3}erf\left(\frac{\left(x_{7}-x_{6}\right)}{d_{3}}\right)+2k_{3}erf\left(\frac{\left(x_{9}-x_{6}\right)}{d_{3}}\right)-k_{1}\left(x_{6}-x_{4}\right)\\ -k_{2}H\left(x_{6}-x_{4}-d_{2}\right){\left(x_{6}-x_{4}-d_{2}\right)}^{3}=0 \end{array}$$

(A7) $$N_{7}:F-k_{3}erf\left(\frac{\left(x_{7}-x_{6}\right)}{d_{3}}\right)-k_{1}\left(x_{7}-x_{5}\right)-k_{2}H\left(x_{7}-x_{5}-d_{2}\right){\left(x_{7}-x_{5}-d_{2}\right)}^{3}=0$$

(A8) $$N_{8}:2k_{3}erf\left(\frac{\left(x_{2}-x_{8}\right)}{d_{3}}\right)-F=0$$

(A9) $$N_{9}:F-2k_{3}erf\left(\frac{\left(x_{9}-x_{6}\right)}{d_{3}}\right)=0$$

The force value between the top end nodes (8th and 9th) should match, as well as between the bottom end nodes (1st and 7th). However, the forces on the top and bottom may not match owing to the strength difference in the network. To obtain F that is employed in the $F_{fiber}$ function, an average was performed.

## References

[1] Purohit P.K., Litvinov R.I., Brown A.E.X., Discher D.E., Weisel J.W. (2011). Protein unfolding accounts for the unusual mechanical behavior of fibrin networks. Acta Biomater..

[2] Brown A.E.X., Litvinov R.I., Discher D.E., Purohit P.K., Weisel J.W. (2009). Multiscale Mechanics of Fibrin Polymer: Gel Stretching with Protein Unfolding and Loss of Water. Science.

[3] Yesudasan S., Wang X., Averett R.D. (2018). Fibrin polymerization simulation using a reactive dissipative particle dynamics method. Biomech. Model. Mechanobiol..

[4] Yesudasan S., Wang X., Averett R.D. (2018). Coarse-grained molecular dynamics simulations of fibrin polymerization: Effects of thrombin concentration on fibrin clot structure. J. Mol. Model..

[5] Münster S., Jawerth L.M., Fabry B., Weitz D.A. (2013). Structure and mechanics of fibrin clots formed under mechanical perturbation. J. Thromb. Haemost..

[6] Piechocka I.K., Bacabac R.G., Potters M., Mackintosh F.C., Koenderink G.H. (2010). Structural Hierarchy Governs Fibrin Gel Mechanics. Biophys. J..

[7] Zhmurov A., Brown A.E.X., Litvinov R.I., Dima R.I., Weisel J.W., Barsegov V. (2011). Mechanism of Fibrin(ogen) Forced Unfolding. Structure.

[8] Arruda E.M., Boyce M.C. (1993). A three-dimensional constitutive model for the large stretch behavior of rubber elastic materials. J. Mech. Phys. Solids.

[9] Brown A.C., Barker T.H. (2013). Fibrin-based biomaterials: Modulation of macroscopic properties through rational design at the molecular level. Acta Biomater..

[10] Mosesson M.W. (2005). Fibrinogen and fibrin structure and functions. J. Thromb. Haemost..

[11] Doolittle R.F. (1984). Fibrinogen and Fibrin. Annu. Rev. Biochem..

[12] Yesudasan S., Averett R.D. (2019). Recent advances in computational modeling of fibrin clot formation: A review. Comput. Boil. Chem..

[13] Badiei N., Sowedan A., Curtis D.J., Brown R., Lawrence M., Campbell A., Sabra A., Evans P., Weisel J., Chernysh I. (2015). Effects of unidirectional flow shear stresses on the formation, fractal microstructure and rigidity of incipient whole blood clots and fibrin gels. Clin. Hemorheol. Microcirc..

[14] Lim B.B., Lee E.H., Sotomayor M., Schulten K. (2008). Molecular Basis of Fibrin Clot Elasticity. Structure.

[15] Averett R.D., Menn B., Lee E.H., Helms C.C., Barker T.H., Guthold M. (2012). A Modular Fibrinogen Model that Captures the Stress-Strain Behavior of Fibrin Fibers. Biophys. J..

[16] Rief M., Gautel M., Oesterhelt F., Fernandez J.M., Gaub H. (1997). Reversible Unfolding of Individual Titin Immunoglobulin Domains by AFM. Science.

[17] Guthold M., Liu W., Sparks E.A., Jawerth L.M., Peng L., Falvo M., Superfine R., Hantgan R.R., Lord S.T. (2007). A Comparison of the Mechanical and Structural Properties of Fibrin Fibers with Other Protein Fibers. Cell Biochem. Biophys..

[18] Kollman J.M., Pandi L., Sawaya M.R., Riley M., Doolittle R.F. (2009). Crystal Structure of Human Fibrinogen. Biochemistry.

[19] Litvinov R.I., Gorkun O.V., Owen S.F., Shuman H., Weisel J.W. (2005). Polymerization of fibrin: Specificity, strength, and stability of knob-hole interactions studied at the single-molecule level. Blood.

[20] Litvinov R.I., Gorkun O., Galanakis D.K., Yakovlev S., Medved L., Shuman H., Weisel J.W. (2006). Polymerization of fibrin: Direct observation and quantification of individual B:b knob-hole interactions. Blood.

[21] Liu W., Carlisle C.R., Sparks E.A., Guthold M. (2010). The mechanical properties of single fibrin fibers. J. Thromb. Haemost..

[22] Levenberg K. (1944). A method for the solution of certain non-linear problems in least squares. Q. Appl. Math..

[23] (1998). Guide, M.U.S.

[24] Powell M.J. (1968). A FORTRAN Subroutine for Solving Systems of Nonlinear Algebraic Equations.

[25] Bouchiat C., Wang M., Allemand J.-F., Strick T.R., Block S., Croquette V. (1999). Estimating the persistence length of a worm-like chain molecule from force-extension measurements. Biophys. J..

[26] Ferry J.D. (1952). The Mechanism of Polymerization of Fibrinogen. Proc. Natl. Acad. Sci. USA.

[27] Chiu S., Stoyan D., Kendall W., Mecke J. (2013). Stochastic Geometry and its Applications.

